# Differential sensitivity to strigolactone signaling and identification of putative D14/KAI2-like receptors in symbiotic *Armillaria*

**DOI:** 10.3389/fmicb.2026.1894528

**Published:** 2026-07-27

**Authors:** Diao Wu, Changfei Zhang, Yan Cheng, Hao Wen, Gang Du, Defu Liu, Menghua Tian

**Affiliations:** 1 Zhaotong Institute of Gastrodia, Zhaotong, Yunnan, China; 2 Yunnan Key Laboratory of Gastrodia and Fungi Symbiotic Biology, Zhaotong University, Zhaotong, Yunnan, China; 3 Yunnan Engineering Research Center of Green Planting and Processing of Gastrodia, Zhaotong University, Zhaotong, Yunnan, China; 4 Key Laboratory of Chemistry in Ethnic Medicinal Resources Ministry of Education, Yunnan Minzu University, Kunming, Yunnan, China

**Keywords:** Armillaria gallica, Armillaria ostoyae, Gastrodia elata, GR24, rhizomorph branching, strigolactone, transcriptome, *α*/*β*-hydrolase receptor

## Abstract

**Introduction:**

Strigolactones (SLs) are carotenoid-derived signaling molecules that play essential roles in plant development and rhizosphere communication, particularly in the establishment of beneficial plant–fungus interactions. Although SL-mediated signaling has been extensively studied in arbuscular mycorrhizal symbioses, its role in the *Gastrodia elata–Armillaria* association remains largely unknown.

**Methods:**

In this study, the *Gastrodia*-symbiotic strain *Armillaria gallica* Arm016 and the non-symbiotic strain *Armillaria ostoyae Arm024* were used to investigate the effects of the synthetic SL analog GR24 on fungal growth, rhizomorph development, and transcriptional responses. To explore the molecular basis of SL perception, three candidate receptor-encoding transcripts were identified in Arm016 through homology-based screening. Domain annotation and structural modeling were performed to characterize their structural features.

**Results:**

GR24 treatment significantly promoted rhizomorph formation in Arm016, increasing rhizomorph numbers by 82.9% compared with the control (*p* < 0.01), whereas only a minor effect was observed in Arm024. Biomass accumulation was enhanced in both strains following GR24 treatment. Transcriptome analysis revealed markedly different responses between the two species. A total of 489 differentially expressed genes (DEGs) were identified in Arm016, compared with only 55 DEGs in Arm024. Functional enrichment analysis indicated that GR24-responsive genes in Arm016 were primarily associated with filamentous growth, metabolic processes, catalytic activity, and transporter functions, suggesting extensive transcriptional reprogramming related to fungal development. In contrast, Arm024 exhibited a comparatively weak transcriptional response. Domain annotation revealed the presence of the conserved α/β-hydrolase (Abhydrolase_1) domain, a hallmark of canonical plant SL receptors. Structural modeling further demonstrated that the candidate protein i2 shares a conserved α/β-hydrolase fold with the Arabidopsis thaliana SL receptor D14 (TM-score = 0.64), supporting its potential role in SL perception.

**Discussion/Conclusion:**

Collectively, these findings demonstrate that symbiotic and non-symbiotic *Armillaria* species exhibit distinct sensitivities to SL signaling and provide preliminary evidence for the existence of D14/KAI2-like SL perception mechanisms in *Armillaria*. This study advances our understanding of chemical communication within the *Gastrodia–Armillaria* symbiosis and provides a foundation for elucidating the molecular mechanisms underlying fungal recruitment and symbiotic establishment.

## Introduction

*Gastrodia elata* Bl. (“Tianma” in Chinese) is an important traditional medicinal orchid that has been widely used in East Asian countries for more than 2,000 years. Its dried tubers are officially recorded in the Chinese Pharmacopoeia and are commonly prescribed for the treatment of neurological disorders, including headache, dizziness, epilepsy, convulsions, and neurodegenerative diseases. Modern pharmacological studies have demonstrated that *G. elata* possesses neuroprotective, antioxidant, anti-inflammatory, anticonvulsant, and cognitive-enhancing activities, largely attributed to bioactive compounds such as gastrodin, parishins, and polysaccharides (Gong et al., 2024; Zhou et al., 2025). Owing to its increasing medicinal demand and high market value, *G. elata* has become one of the most economically important medicinal crops in southwestern China, particularly in Yunnan, Guizhou, Sichuan, and Hubei provinces (Wu et al., 2023).

Unlike most green plants, *G. elata* is a fully mycoheterotrophic orchid that lacks chlorophyll and depends entirely on fungal partners for carbon and nutrient acquisition throughout its life cycle. During seed germination, protocorm development, and tuber enlargement, *G. elata* establishes intimate associations with different fungal partners, among which species of *Armillaria* play indispensable roles during vegetative growth and tuber production (Cha and Igarashi, 1995; Suetsugu et al., 2020). Therefore, understanding the molecular basis of fungal recognition and symbiotic establishment is essential for improving the cultivation efficiency and sustainable production of *G. elata*.

Species of *Armillaria* are ecologically diverse fungi exhibiting saprotrophic, pathogenic, or symbiotic lifestyles. In its saprotrophic stage, it is capable of efficiently degrading major plant components, including lignin, cellulose, hemicellulose, and pectin (Sipos et al., 2017). To date, more than 40 biological species have been recognized worldwide, and approximately 15 species have been reported in China (Guo et al., 2016; Heinzelmann et al., 2019). However, only a limited number of species can form stable and productive symbiotic relationships with *G. elata*. Specifically, during tuber formation, the fungal rhizomorphs invade *G. elata* tubers and are digested, thereby serving as a vital nutrient source for its growth and development (Cha and Igarashi, 1995; Suetsugu et al., 2020). Previous studies have demonstrated that different *Armillaria* species vary substantially in colonization ability, nutrient transfer efficiency, and their capacity to promote tuber yield and quality (Wang et al., 2023). In terms of pathogenicity, *A. ostoyae*, *A. mellea*, and *A. borealis* are considered virulent pathogens, whereas *A. gallica*, *A. sinapina*, *A. gemina*, *A. calvescens*, *A. cepistipes*, and *A. nabsnona* are regarded as less virulent (Caballero et al., 2023; Morrison, 2004; Prospero et al., 2004). Interestingly, weakly pathogenic species with predominantly saprotrophic characteristics, such as *A. gallica*, generally exhibit stronger compatibility with *G. elata* than highly virulent species, including *A. ostoyae* and *A. mellea* (Guo et al., 2016; Caballero et al., 2023). Nevertheless, the signaling mechanisms underlying the selective establishment of these symbiotic interactions remain poorly understood.

Strigolactones (SLs) are plant-derived carotenoid derivatives that function both as endogenous regulators of plant development and as rhizosphere signaling molecules (Pandey et al., 2016). Since their discovery as germination stimulants for parasitic plants, SLs have been shown to regulate shoot branching, root architecture, leaf senescence, stress adaptation, and nutrient acquisition (Al-Babili and Bouwmeester, 2015; Waters et al., 2017). Current studies indicate that SLs can be secreted into the rhizosphere to influence the growth of soil microorganisms surrounding the roots (Lanfranco et al., 2018; Al-Babili and Bouwmeester, 2015). Notably, they play a crucial role in facilitating the symbiosis between plants and arbuscular mycorrhizal fungi (AMF) as well as nitrogen-fixing rhizobia (Waters et al., 2017). One of the most important ecological functions of SLs is their involvement in plant responses to phosphate (Pi) deficiency. Under low-phosphate conditions, plants markedly upregulate SL biosynthesis and secretion into the rhizosphere, where SLs stimulate the growth and branching of beneficial microorganisms, thereby enhancing nutrient acquisition and improving plant fitness (Naseer et al., 2024). Recent studies have highlighted the central role of SLs in mediating plant–fungus interactions. In arbuscular mycorrhizal symbioses, SLs secreted by plant roots induce extensive hyphal branching, mitochondrial activation, and transcriptional reprogramming in fungal partners, thereby promoting colonization and phosphorus uptake (Akiyama et al., 2005; Salvioli et al., 2016). Furthermore, phosphorus starvation has been shown to coordinately regulate SL biosynthesis and symbiotic signaling pathways, establishing a mechanistic link between nutrient sensing and microbial recruitment (Naseer et al., 2024). Because *G. elata* is completely dependent on fungal-derived nutrients, the regulation of fungal growth and nutrient transfer by SL signaling may represent an evolutionarily important mechanism facilitating the establishment and maintenance of the *Gastrodia–Armillaria* symbiosis.

Although the biological functions of SLs in arbuscular mycorrhizal fungi have been extensively investigated, relatively little is known about their effects on basidiomycete fungi, particularly *Armillaria* species. Dor et al. (2011) demonstrated that fungal responses to GR24, a synthetic SL analog, vary considerably among species. In contrast, Huang et al. (2019) found that GR24 significantly promoted rhizomorph branching and elongation in *A. gallica*. Because rhizomorphs are responsible for nutrient transport, host colonization, and symbiotic establishment, enhanced rhizomorph development may directly influence the productivity of *G. elata*. However, the molecular mechanisms underlying SL perception and signaling in *Armillaria* remain unknown, and the basis for differential responsiveness among symbiotic and non-symbiotic species has not been explored.

In the present study, the *Gastrodia*-symbiotic strain *A. gallica* and the non-symbiotic strain *A. ostoyae* were selected as model organisms. Using the synthetic strigolactone analog GR24, we systematically investigated the effects of SL signaling on rhizomorph branching, mycelial biomass accumulation, and transcriptomic responses in both strains. In addition, candidate SL receptor proteins in Arm016 were identified and analyzed through bioinformatic and structural approaches. This study provides new insights into the molecular mechanisms underlying SL perception in *Armillaria* and contributes to a deeper understanding of chemical communication and symbiotic regulation within the *Gastrodia–Armillaria* interaction system.

## Materials and methods

### Fungal materials and culture conditions

The *Gastrodia*-symbiotic fungus Arm016 was isolated from a *Gastrodia elata* cultivation base in Yiliang County, Zhaotong City, Yunnan Province, China. This strain was obtained from soil samples by our laboratory in 2023 and is currently preserved at the cultivation base in Yiliang County. The non-symbiotic fungus Arm024 was originally obtained from the Beijing Beina Chuanglian Biotechnology Research Institute, China. Prior to the experiments, both strains were verified for their symbiotic capabilities: The *Gastrodia*-symbiotic fungus Arm016 was confirmed to establish a symbiotic relationship with *G. elata* and induce the formation of flowering stems, whereas the non-symbiotic fungus Arm024 was unable to do so.

*A. gallica* Arm016 and *A. ostoyae* Arm024 were routinely cultured on potato dextrose agar (PDA) medium at 25 °C. PDA medium was prepared with 200 g potato, 20 g glucose (Solarbio, G8150), 5 g KH₂PO₄ (Sigma-Aldrich, P5379), 1.5 g MgSO₄·7H₂O (Sinopharm, S63140500GF), and 15 g agar (Solarbio, P8931) per liter of deionized water. Cultures were maintained by regular subculturing on fresh PDA plates and stored at 4 °C until use.

### Strain identification

The two *Armillaria* strains were identified based on sequence analysis of the translation elongation factor 1-alpha (*tef1-α*) gene amplified using primers EF595F and EF1160R. The resulting sequences were subjected to BLAST searches against the NCBI GenBank database. Strain Arm016 showed the highest similarity to *Armillaria gallica* (KM205261.1), with 100% sequence identity and 94% query coverage, whereas strain Arm024 showed the highest similarity to *Armillaria ostoyae* (KT822428.1), with 100% sequence identity and 100% query coverage. Both matches exhibited E-values below 1 × 10^−100^. Combined with morphological observations, these results confirmed the identities of the two strains as *A. gallica* and *A. ostoyae*, respectively.

### GR24 treatment and rhizomorph growth analysis

Based on Yuan et al. (2018), the working concentration of the strigolactone analog GR24 (Beijing Solarbio Science & Technology Co., Ltd., Cat. No. IS3390) was set at 1 mg/L. Under sterile conditions, 100 μL of GR24 solution (1 mg/L) was added dropwise to the surface of PDA solid medium and spread evenly for the experimental groups, while an equal volume of sterile water was added for the control groups. For both the symbiotic *A. gallica* (Arm016) and the non-symbiotic *A. ostoyae* (Arm024), independent experimental and control groups were established: Arm016 + GR24 (experimental) and Arm016 + sterile water (control); Arm024 + GR24 (experimental) and Arm024 + sterile water (control). Each group was performed in triplicate to ensure data reliability. For each treatment, an agar plug (approximately 0.5 × 0.5 × 0.5 cm) containing actively growing mycelium from the margin of Arm016 or Arm024 colonies was transferred to the center of a Petri dish. Cultures were incubated at 25 °C under dark conditions. After 7 days of incubation, rhizomorph growth was assessed and documented. Rhizomorph morphology was photographed using a digital camera (Canon EOS 80D), and the total number of rhizomorph branches produced by each colony was recorded.

### Effect of SLs on biomass

Actively growing mycelial plugs (6 mm in diameter) were excised from the colony margins of *A. gallica* Arm016 and *A. ostoyae* Arm024 grown on PDA plates and inoculated into 500-mL Erlenmeyer flasks containing 200 mL of liquid seed medium. The cultures were incubated at 25 °C on a rotary shaker at 180 rpm for 7 days to prepare fungal seed cultures.

Subsequently, Arm016 and Arm024 strains were inoculated into liquid PDA medium supplemented with GR24 to a final concentration of 0.1 mg/L. Control groups without GR24 were established in parallel. Each treatment included three independent biological replicates. All fermentation cultures were incubated at 25 °C and 180 r/min for 15 days. During the incubation period, the color of the broth and the growth of mycelial pellets were observed and recorded.

At the end of the fermentation, the broth was filtered under suction to harvest the mycelia. The collected mycelia were washed three times with sterile water and dried at 60 °C to a constant weight. The mycelial biomass was calculated using the following Equation 1:


$$\begin{array}{l} \text{Mycelial Biomass}\;(g/L)=(\mathrm{Dry}\;\text{Weight of Mycelia}\;(g)\;)/\\ \left(\text{Volume of Fermentation Broth}\;(L)\right) \end{array}$$ (1)

### Transcriptome analysis

To investigate the effects of strigolactones (SLs) on gene expression in the two Armillaria strains (Arm016 and Arm024), fresh mycelial pellets were harvested from liquid cultures, immediately frozen in liquid nitrogen, and subsequently stored at −80 °C until further use. Samples were subsequently sent to Novogene Co., Ltd. (Beijing, China)^1^ for RNA extraction, library construction, and sequencing. Briefly, total RNA was extracted from fresh mycelia using the RNeasy Mini Kit (Qiagen, Hilden, Germany) following the manufacturer’s instructions, and genomic DNA contamination was removed using DNase I (Invitrogen, USA). RNA degradation and purity were monitored using 1% agarose gel electrophoresis. The purity and concentration of total RNA were determined using a NanoPhotometer® (Implen, Germany) and a Qubit® RNA Assay Kit (Agilent Technologies, USA), respectively. Transcriptome sequencing was performed on 3 μg of total RNA per sample. PCR products were purified using the AMPure XP system, and library quality was assessed using the Agilent Bioanalyzer 2,100 system (USA). Indexed coding samples were clustered on the cBot Cluster Generation System using the TruSeq PE Cluster Kit v3-cBot-HS (Illumina, USA) according to the manufacturer’s protocol. Following cluster generation, library preparation was sequenced on an Illumina HiSeq platform to generate paired-end reads of 125 bp or 150 bp. Raw data have been uploaded to the National Center for Biotechnology Information (NCBI) database under SRA project accession number PRJNA759758.

### *De novo* assembly by trinity

The clean reads were generated by filtering low-quality sequences, unknown reads larger than 5%, and adapter sequences with Trimmomatic v0.36. Then, the clean reads were assembled by using the Trinity program to acquire unique consensus unigenes. The assembled transcripts were quantitatively expressed by the RESM (Reads Per Kilobase per Million mapped reads) method. What’s more, the gene-coding regions of UniGene sequences were predicted using TransDecoder v5.3.04.2.7.

### Differential gene expression analysis

The read count matrix was obtained by using StringTie v2.1.0 for expression qualification. The read count matrix was imported into R 3.6.3. DESeq2 of the R package was used for differential gene analysis, with an FDR < 0.05 and |log2FC| > 2. Then, for functional annotation and classification, all transcripts and their corresponding genes were compared by emapper v2.1.3. The visualization of GO functional analysis results was performed by using WEGO.^2^

### Strigolactone receptor prediction analysis

Protein sequences of known strigolactone receptors were downloaded from the UniProt database. These sequences were subsequently used as queries to perform BLAST searches against the predicted protein sequences derived from the transcriptomes of the sequenced *Armillaria* strains. Transcripts with an E-value ≤ 1 × 10^−5^ and detectable expression in the transcriptome datasets were retained for further analysis. Finally, the candidate sequences were subjected to conserved domain verification using the Pfam database to confirm the presence of characteristic protein domains associated with strigolactone receptor function.

The predicted sequences were uploaded to AlphaFold2 for structural prediction. The results were then visualized using the Mol3D Viewer tool on the RCSB official website.^3^ Finally, pairwise structural alignment was performed using the Pairwise Structure Alignment tool to compare the three-dimensional structures of the proteins.

### Data processing and statistical analysis

All experiments were performed in at least three replicates. The results were expressed as the mean ± standard error (SE). Data were statistically processed using Microsoft Excel 2020 to calculate the mean values. GraphPad Prism 9.5 was used for graphing, calculating the SE, and performing significance analysis (one-way ANOVA).

## Results

### Effects of GR24 on rhizomorph formation in *Armillaria* strains

**Figure 1:**
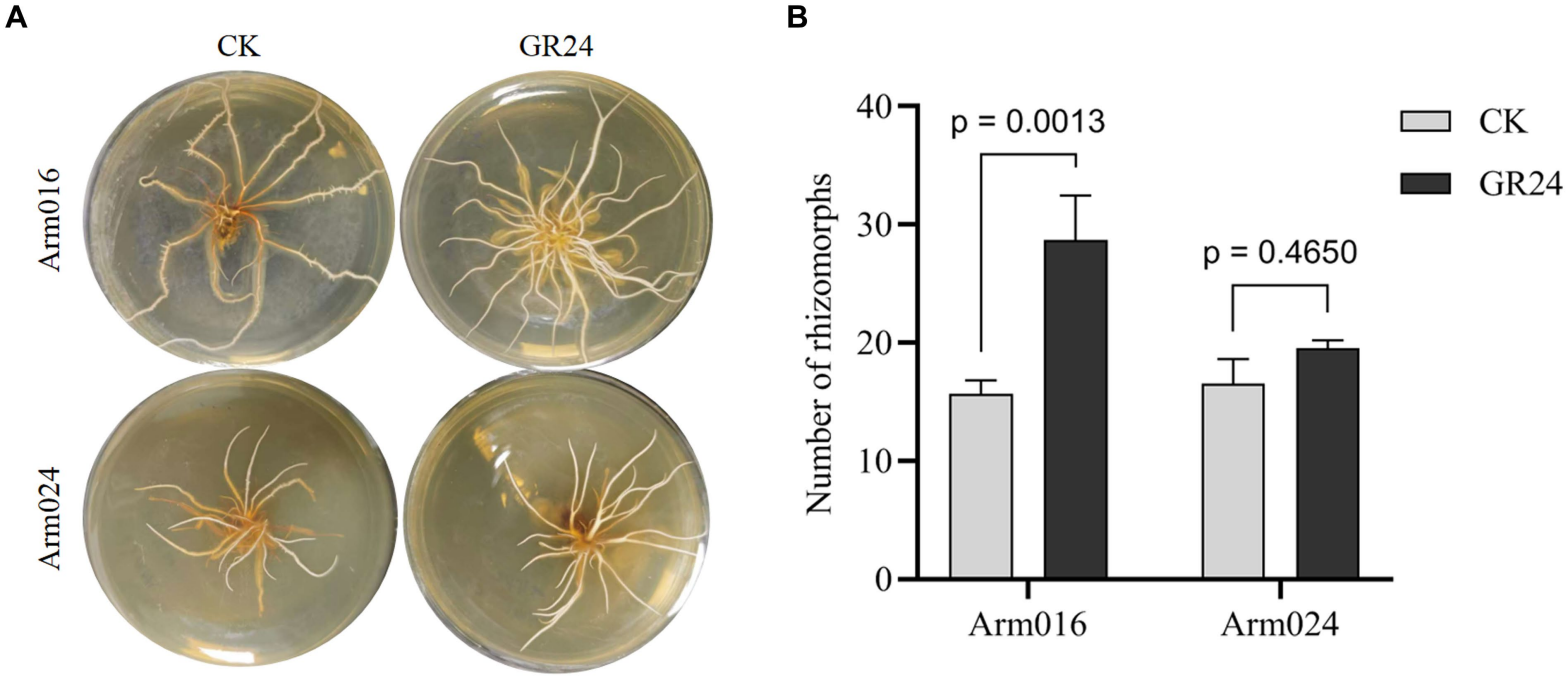
Morphology and number of rhizomorphs in *A. gallica* Arm016 and *A. ostoyae* Arm024 under strigolactone treatment. **(A)** Colony morphology on PDA plates. **(B)** Statistical analysis of rhizomorph number.

Significant differences in rhizomorph formation were observed between the two *Armillaria* strains following GR24 treatment. In the symbiotic strain Arm016, GR24 significantly stimulated rhizomorph development, with the average number of rhizomorphs reaching 28.7 ± 3.8, representing an 82.9% increase compared with the control group (15.7 ± 1.2). This difference was highly significant (*p <* 0.01). In contrast, GR24 treatment had no significant effect on rhizomorph formation in the non-symbiotic strain Arm024. The mean numbers of rhizomorphs in the GR24-treated and control groups were 19.5 ± 0.5 and 16.5 ± 1.5, respectively, corresponding to an 18.2% increase that was not statistically significant (*p* > 0.05) (Figure 1). These results indicate that the response to strigolactone signaling differs markedly between the two *Armillaria* species, with the symbiotic strain Arm016 exhibiting a substantially stronger rhizomorph developmental response to GR24 than the non-symbiotic strain Arm024.

### Effects of GR24 on mycelial biomass accumulation in *Armillaria* strains

**Figure 2:**
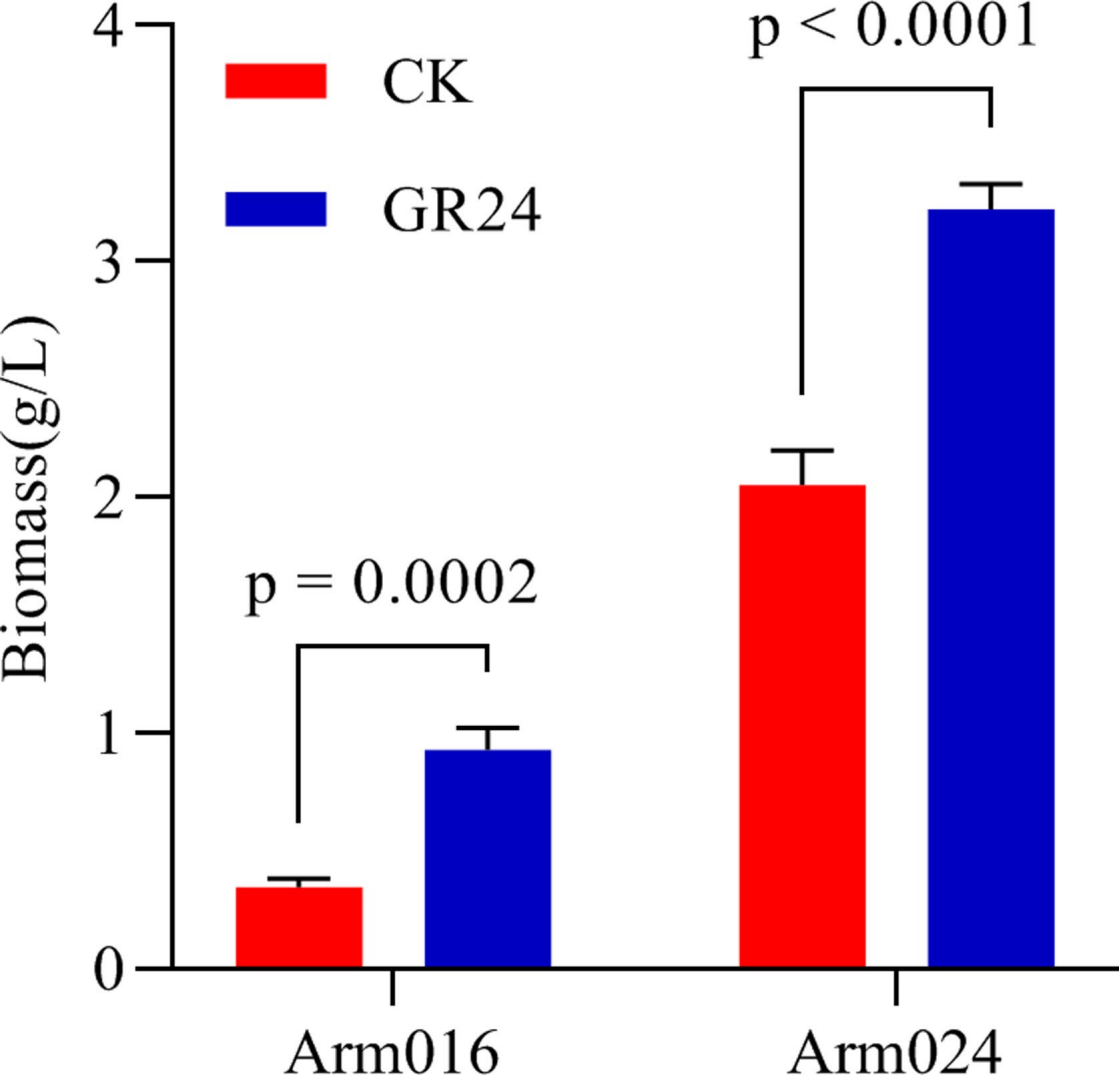
Effects of strigolactone on the biomass of *A. gallica* Arm016 and *A. ostoyae* Arm024.

As shown in Figure 2, GR24 treatment significantly increased the mycelial biomass of both *A. gallica* Arm016 and *A. ostoyae* Arm024 compared with their respective control groups (*p <* 0.01). These results demonstrate that the strigolactone analog GR24 effectively promotes biomass accumulation in both *Armillaria* strains, suggesting that strigolactone signaling may positively regulate fungal growth and mycelial development.

### Evaluation of transcriptome sequencing data

Transcriptome sequencing was conducted on three biological replicates of both the control (CK) and GR24-treated groups of the symbiotic strain *A. gallica* Arm016 and the non-symbiotic strain *A. ostoyae* Arm024. Sequencing generated between 13.65 and 18.83 million raw reads and 12.70 to 18.02 million clean reads for Arm016. For Arm024, 14.59 to 18.99 million raw reads and 13.58 to 17.83 million clean reads were obtained after quality filtering.

Based on the high-quality reads, *de novo* transcriptome assemblies were generated for both strains. The assembled transcriptome of the symbiotic strain Arm016 contained 129,914 annotated genes and 234,251 transcripts, with a GC content of 49.69% and a BUSCO completeness score of 74.2%. In comparison, the transcriptome of the non-symbiotic strain Arm024 comprised 68,279 annotated genes and 168,486 transcripts, with a GC content of 50.09% and a BUSCO completeness score of 76.3% (Table 1).

**Table 1:** Statistics of transcriptome assembly results.

Strain ID	Number of genes	Number of transcripts	GC content (%)	BUSCO completeness (%)
Arm016	129,914	234,251	49.69%	74.2%
Arm024	68,279	168,486	50.09%	76.3%

### Differential gene expression analysis

**Figure 3:**
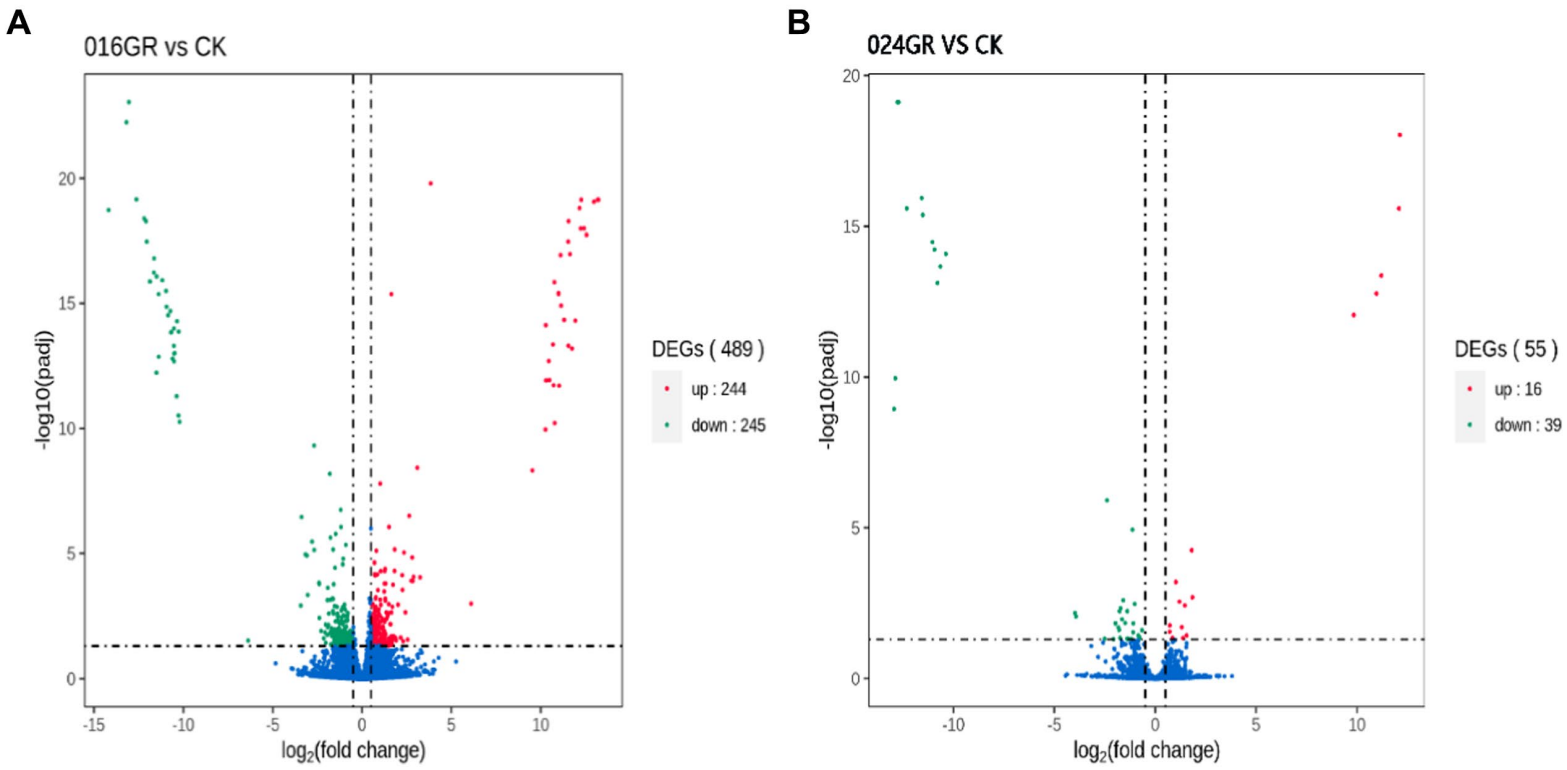
Volcano plots showing differentially expressed genes in *A. gallica* Arm016 and *A. ostoyae* Arm024 treated with GR24: **(A)** 016GR vs. 016CK; **(B)** 024GR vs. 024CK.

The results of differential gene expression analysis are presented in Figure 3. In the symbiotic strain Arm016, GR24 treatment induced substantial transcriptomic reprogramming compared with the control (CK). A total of 489 differentially expressed genes (DEGs) were identified, comprising 244 upregulated and 245 downregulated genes (Figure 3A). The relatively large number of DEGs indicates that the symbiotic strain Arm016 exhibits a pronounced molecular response to strigolactone signaling, which is consistent with the observed promotion of rhizomorph formation and biomass accumulation following GR24 treatment.

In contrast, only 55 DEGs were detected in the non-symbiotic strain Arm024 under GR24 treatment, including 16 upregulated and 39 downregulated genes (Figure 3B). Compared with the symbiotic strain Arm016, the non-symbiotic strain Arm024 displayed a markedly lower number of DEGs, with most genes distributed near the center of the volcano plot. This indicates relatively small fold changes and limited transcriptional responsiveness. These results suggest that, at the transcriptomic level, the non-symbiotic strain Arm024 exhibits a substantially weaker response to GR24, which aligns with the phenotypic observations showing only minor effects of GR24 on rhizomorph development.

Overall, the differential expression profiles reveal distinct transcriptional responses to strigolactone signaling between the two *Armillaria* species. The symbiotic strain Arm016 showed a much stronger transcriptional response than the non-symbiotic strain Arm024, suggesting that its enhanced sensitivity to strigolactones may be associated with symbiotic adaptation to *Gastrodia elata*.

### GO enrichment analysis of DEGs

**Figure 4:**
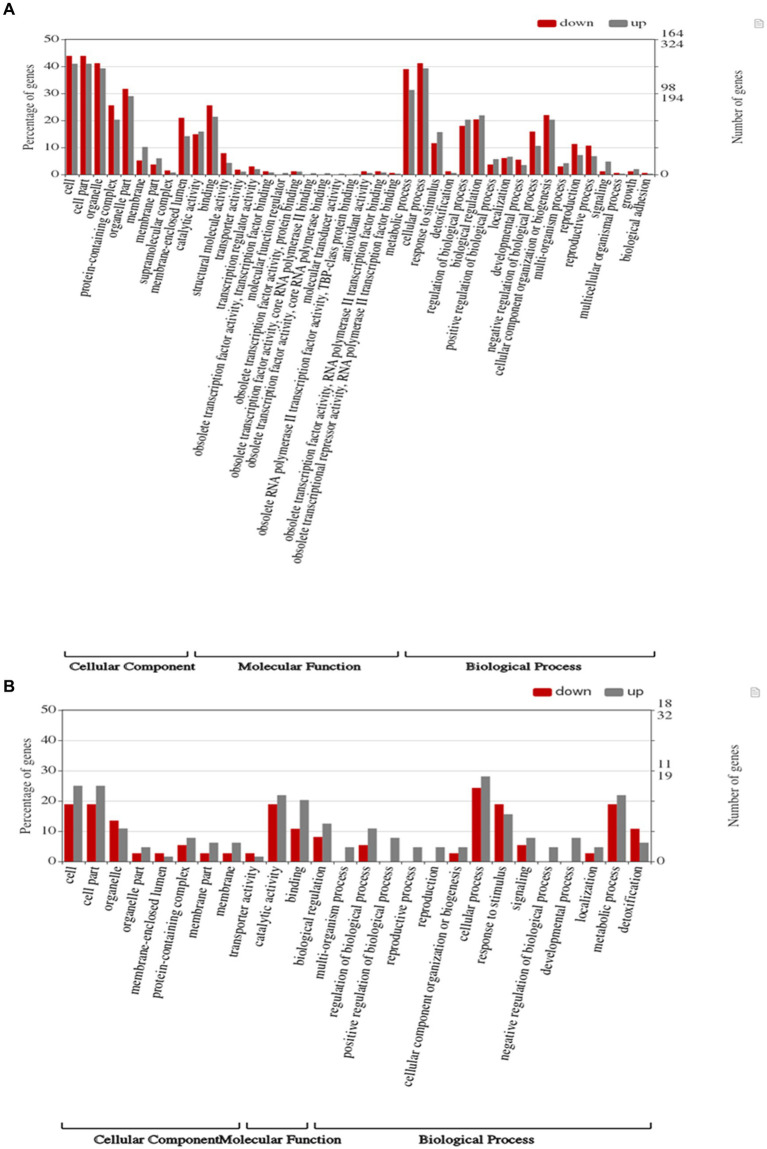
GO enrichment analysis of DEGs: **(A)** *A. gallica* Arm016; **(B)** *A. ostoyae* Arm024.

To elucidate the molecular mechanisms underlying the strigolactone (SL)-mediated promotion of growth and branching in *Armillaria* species, Gene Ontology (GO) enrichment analysis was performed on the differentially expressed genes (DEGs). As shown in Figure 4, these DEGs were categorized into three main classes: molecular function (MF), cellular component (CC), and biological process (BP).

In terms of cellular components (CC), the DEGs in both the symbiotic strain Arm016 and the non-symbiotic strain Arm024 were predominantly enriched in the “cell growth” (GO:0016049) and “filamentous growth” (GO:0030447) terms. Notably, the number of upregulated genes in the symbiotic strain Arm016 significantly exceeded that of downregulated genes. This pronounced upregulation suggests that the fungus is massively synthesizing novel cellular structures, characterized by the rapid assembly of cell membranes, organelles, and protein complexes. This structural expansion provides the necessary cytological foundation for the observed increase in biomass and the substantial proliferation of rhizomorphs.

Regarding molecular function (MF), the DEGs were significantly enriched in catalytic activity, transporter activity, and small molecule binding. Furthermore, in the biological process (BP) category, the DEGs were primarily associated with metabolic processes, biological regulation, and responses to stimuli, highlighting the extensive regulatory network activated by SL signaling.

### Venn diagram analysis of DEGs

**Figure 5:**
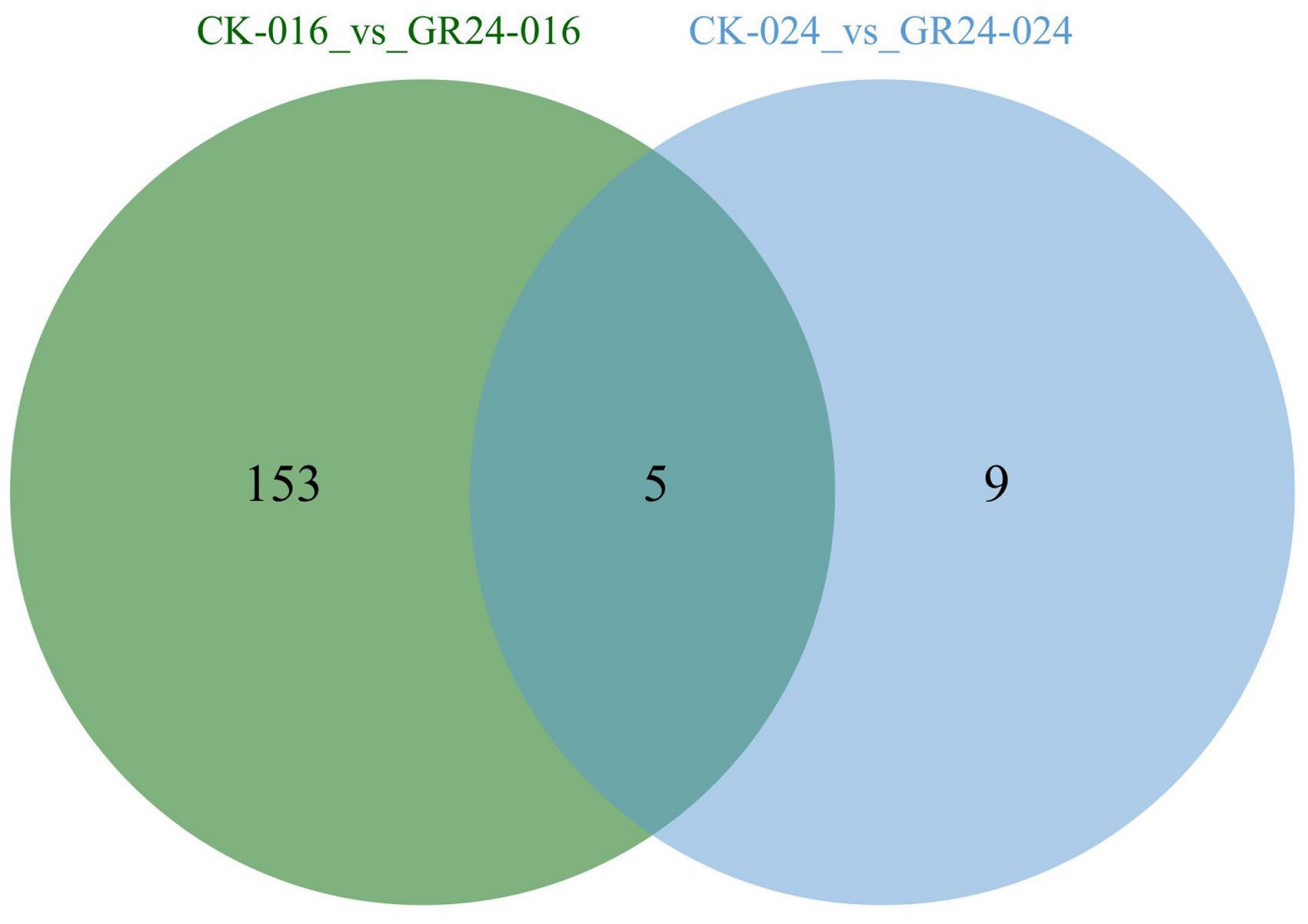
Venn diagram of differentially expressed genes.

A Venn diagram analysis was conducted to compare the functional annotations of upregulated genes in the symbiotic *A. gallica* strain Arm016 and the non-symbiotic *A. ostoyae* strain Arm024 following GR24 treatment (Figure 5). The results revealed marked differences in their transcriptional response patterns. A total of 153 unique functional annotation terms were identified in the symbiotic strain Arm016, whereas only 9 unique functional annotations were detected in the non-symbiotic strain Arm024. In addition, the two strains shared only five common functional categories. The substantially greater number of Arm016-specific functional annotations suggests that GR24 induced a broader and more diverse transcriptional response in the symbiotic strain than in the non-symbiotic strain. These findings further support the hypothesis that the symbiotic strain Arm016 exhibits a stronger responsiveness to strigolactone signaling, a characteristic that may have evolved through its long-term adaptation to symbiosis with *Gastrodia elata*.

Table 2 lists the top 10 unique gene functional categories with the highest annotations, as well as all shared categories. Notably, proteins of the *α*/*β*-hydrolase fold family were significantly up-regulated in the symbiotic strain Arm016. Given that known strigolactone receptors in plants are α/β-hydrolases encoded by D14/KAI2 genes—which possess dual functions of perception and hydrolysis—this finding suggests the existence of potential D14/KAI2-like strigolactone receptors in Arm016 to respond to SLs signaling.

**Table 2:** Up-regulated gene functions in *Armillaria* under the influence of strigolactones (partial).

No.	Arm016	Arm024	Common
1	Alpha/beta hydrolase fold family	Non-heme compound in morphine synthesis N-terminal process	Sulfate adenylyltransferase
2	Eukaryotic ribosomal protein family	DapA family	FAD-dependent oxidoreductase
3	Glycosyl hydrolase family 16	Hal2 nucleotidase	Major facilitator superfamily
4	Anti-apoptotic protein Bax1	Coproporphyrinogen III oxidase	P-loop containing nucleoside triphosphate hydrolase
5	Protein of unknown function	Zinc-binding dehydrogenase	Cytochrome P450
6	Protein containing permanent domain	WD40 repeat-like protein	–
7	Translation initiation factor 2	WH1 domain	–
8	Zinc finger motif	G protein alpha subunit	–
9	Cyclin-binding protein	Iron permease FTR1 family	–
10	Peptidyl-prolyl cis-trans isomerase	–	–

In contrast, no significant enrichment of α/β-hydrolase functions was detected in the non-symbiotic strain Arm024. However, its unique up-regulated genes included the “G protein alpha subunit,” a key component of the cAMP signaling pathway. This indicates that non-symbiotic strain Arm024 might lack a direct SLs receptor and instead relies on G protein-coupled or downstream kinase cascade-mediated signal transduction pathways to perceive SLs stimulation.

### Prediction of candidate strigolactone receptors in the symbiotic strain *A. gallica* Arm016

Through sequence homology alignment and functional annotation screening, this study initially identified three candidate strigolactone receptor protein-encoding transcripts in the symbiotic strain Arm016: TRINITY_DN3825_c0_g2_i2 (i2), TRINITY_DN3825_c0_g2_i3 (i3), and TRINITY_DN5314_c0_g1_i5 (i5).

To validate the structural characteristics of these candidate proteins, the predicted protein sequences were submitted to the Pfam database for domain annotation. Simultaneously, all confirmed known protein sequences from the UniProt database were annotated as a reference group to compare the conservation of domain composition between the two.

The analysis results showed that the candidate proteins in the symbiotic strain Arm016 contain the characteristic domain of the *Abhydrolase* (*α*/*β*-hydrolase) family (Table 3). The conservation of this domain suggests that the identified transcripts may possess functions similar to D14/KAI2, indicating they are potential strigolactone receptors in the symbiotic strain Arm016.

**Table 3:** Strigolactone receptor proteins and domains.

Receptor protein/domain source	Name	Protein Domain	Domain Schematic	References
Known receptor proteins and domains	Q9SQR3	*Abhydrolase_6*	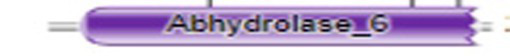	Yao et al. (2016) and Chevalier et al. (2014)
	Q10QA5	*Abhydrolase_6*	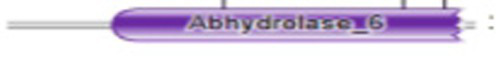	Kagiyama et al. (2013)
	A0A109QYD3	*Abhydrolase_6*	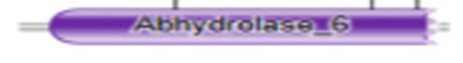	de Saint Germain et al. (2016)
	G7JIK2	*PK_Tyr_Ser-Thr*	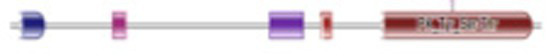	Müller et al. (2019)
	Q6L545	*Abhydrolase_3*	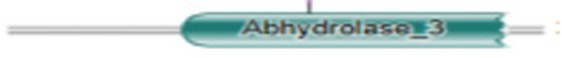	Ueguchi-Tanaka et al. (2005)
	Q96524	*FAD_binding_7*	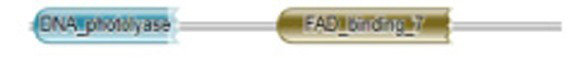	Özgür and Sancar (2006)
	Q43125	*FAD_binding_7*	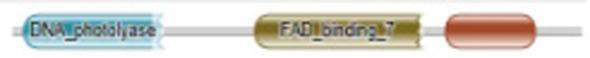	Özgür and Sancar (2006)
	Q9SIM9	*F-box_5*	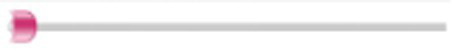	Zhang et al. (2016)
	Q7G7J6	*GRAS*	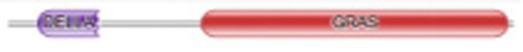	Dai and Xue (2010)
	Q10J20	*Abhydrolase_6*	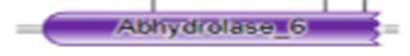	Gutjahr et al. (2015)
Predicted receptor proteins and domains in Arm016	TRINITY_DN3825_c0_g2_i2	*Abhydrolase_1*	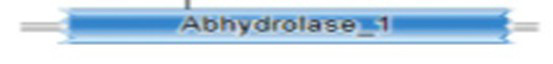	–
	TRINITY_DN3825_c0_g2_i3	*Abhydrolase_1*	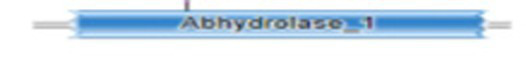	–
	TRINITY_DN5314_c0_g1_i5	*Abhydrolase_1*	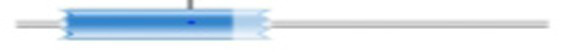	–

### Structural alignment of strigolactone receptors

To investigate the three-dimensional structural characteristics and potential functional conservation of strigolactone receptors, the candidate protein i2 identified from the symbiotic strain Arm016 was subjected to structural prediction using AlphaFold2. The resulting model was subsequently compared with the crystal structure of the well-characterized strigolactone receptor D14 from *Arabidopsis thaliana* (PDB ID: 4IH4).

**Figure 6:**
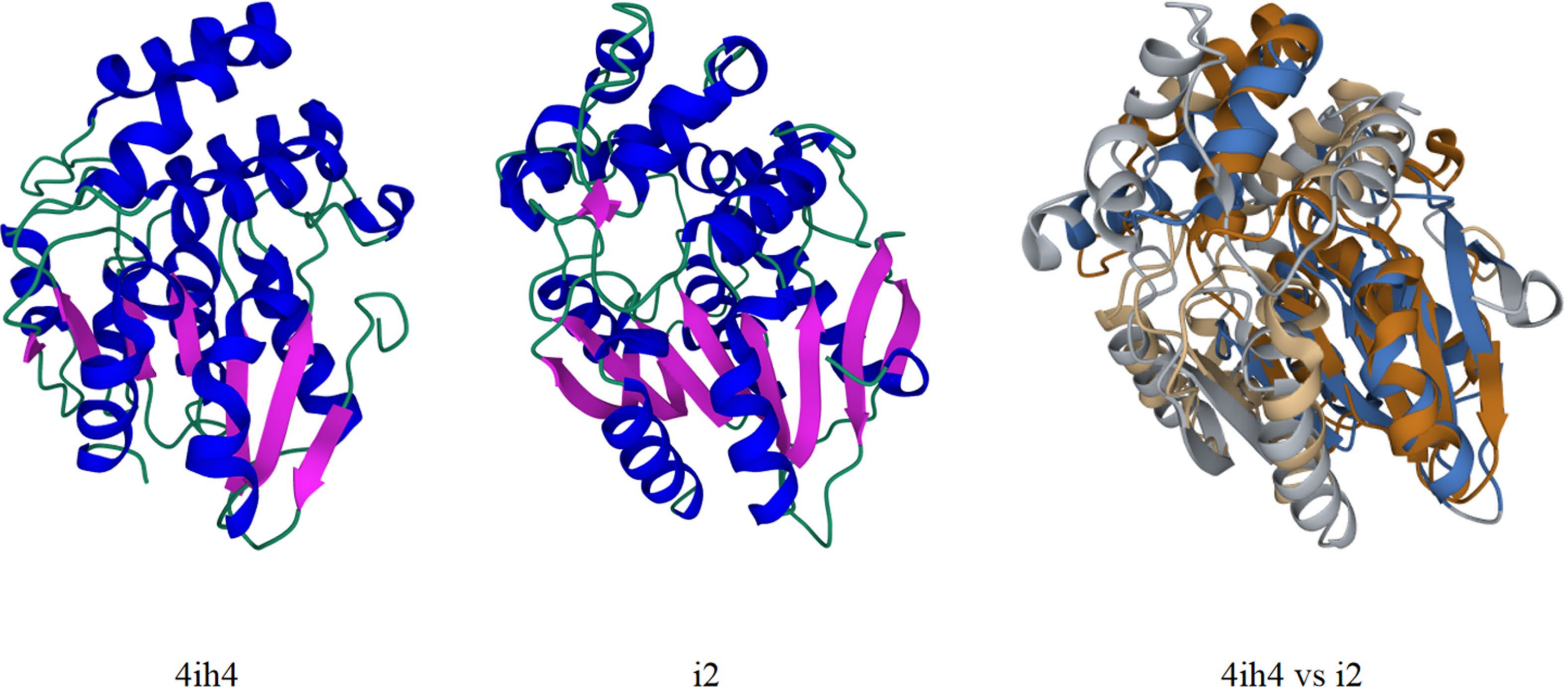
Strigolactone receptor structures. Blue regions represent α-helices, and purple regions represent β-sheets. Dark colors indicate high alignment matching, while gray regions indicate low alignment matching.

As shown in Figure 6, the predicted i2 protein exhibited a high degree of similarity to the reference protein 4IH4 in its overall three-dimensional architecture. Structural alignment revealed a sequence similarity (SS) of 30% and a template modeling score (TM-score) of 0.64. Since TM-scores >0.5 generally indicate that two proteins share the same or a highly similar overall fold, these results suggest that i2 possesses a conserved structural framework comparable to that of known strigolactone receptors.

Further structural analysis demonstrated that both i2 and the *Arabidopsis* D14 receptor adopt the characteristic *α*/*β*-hydrolase fold and contain nine conserved β-strands. The high degree of structural conservation between these proteins supports the classification of i2 as a member of the α/β-hydrolase superfamily. Given that canonical strigolactone receptors belong to this protein family, the observed structural similarity suggests that i2 may represent a putative strigolactone receptor in *Armillaria* species and could participate in strigolactone perception and downstream signal transduction. However, its precise biological function remains to be experimentally validated.

## Discussion

Strigolactones (SLs) were initially identified as germination stimulants for parasitic weeds of the genus *Striga* (Cook et al., 1966). Subsequent studies demonstrated that SLs function as multifunctional signaling molecules regulating plant architecture, nutrient acquisition, abiotic stress responses, and rhizosphere communication (Al-Babili and Bouwmeester, 2015; Waters et al., 2017). One of the most extensively studied ecological functions of SLs is their role in establishing beneficial plant–microbe interactions, particularly with arbuscular mycorrhizal fungi (AMF). Root-derived SLs stimulate hyphal branching, activate mitochondrial metabolism, and promote fungal colonization, thereby enhancing phosphorus acquisition under nutrient-limited conditions (Akiyama, Matsuzaki, and Hayashi 2005; Besserer et al., 2006; Salvioli et al., 2016).

Compared with AMF, however, the role of SLs in basidiomycete fungi remains poorly understood. Previous studies have reported highly variable responses of different fungal taxa to GR24. Dor et al. (2011) found that GR24 stimulated hyphal branching in several phytopathogenic fungi, whereas Liu et al. (2025) observed that different Armillaria species exhibited varying responses to GR24. These findings suggest that fungal responsiveness to SLs is species-specific and may depend on the ecological lifestyle of the fungus. Consistent with this hypothesis, our results demonstrated a striking difference between the Gastrodia-symbiotic strain *A. gallica* Arm016 and the non-symbiotic strain *A. ostoyae* Arm024. GR24 significantly promoted rhizomorph formation in Arm016 but had only a limited effect on Arm024, indicating that SL responsiveness differs substantially among *Armillaria* species.

Rhizomorphs are the primary structures responsible for host colonization, nutrient transport, and long-distance growth in *Armillaria* species (Heinzelmann et al., 2019). In the *Gastrodia–Armillaria* symbiosis, rhizomorphs directly determine fungal colonization efficiency and nutrient delivery to developing tubers (Deng, 2018). Therefore, the pronounced increase in rhizomorph branching observed in Arm016 following GR24 treatment may have important ecological implications. Enhanced rhizomorph development could improve host-fungus contact frequency, nutrient transport capacity, and ultimately symbiotic efficiency. This interpretation is consistent with previous observations that highly compatible *Armillaria* strains typically exhibit stronger rhizomorph growth and confer greater yield benefits to *G. elata* cultivation systems (Guo et al., 2016; Wang et al., 2023).

Transcriptome analysis further revealed that Arm016 exhibited a substantially stronger molecular response to GR24 than Arm024. A total of 489 DEGs were identified in Arm016, nearly nine times more than the 55 DEGs detected in Arm024. GO enrichment analysis indicated that many of these genes were associated with cell growth, filamentous growth, transporter activity, catalytic activity, and metabolic processes. These transcriptional changes are consistent with the observed increases in biomass accumulation and rhizomorph branching. Similar transcriptional reprogramming has been reported in AMF exposed to SLs, where genes involved in respiration, energy metabolism, and hyphal development are rapidly activated (Besserer et al., 2006; Salvioli et al., 2016). Our findings suggest that although *Armillaria* belongs to Basidiomycota rather than Glomeromycota, SL signaling may similarly regulate fungal growth through coordinated activation of metabolic and developmental pathways.

One of the most intriguing findings of this study was the enrichment of *α*/*β*-hydrolase-related genes in the symbiotic strain Arm016. In plants, canonical SL receptors such as D14 and KAI2 belong to the α/β-hydrolase superfamily and function through ligand-dependent hydrolysis and signal transduction (Yao et al., 2016; Waters et al., 2017). Until recently, direct evidence for fungal SL receptors was lacking. Wang et al. (2025) reported that CpD14, an α/β-hydrolase identified in the phytopathogenic fungus *Cryphonectria parasitica*, exhibits both SL-binding and hydrolytic activities, providing the first experimental evidence that fungi may possess D14-like SL perception mechanisms. In the present study, three candidate α/β-hydrolase proteins were identified in Arm016, and structural analysis demonstrated that candidate protein i2 shares the characteristic α/β-hydrolase fold and conserved tertiary architecture of the *Arabidopsis* D14 receptor. Although these findings do not conclusively establish receptor function, they provide preliminary evidence supporting the existence of D14/KAI2-like SL perception systems in *Armillaria*.

Interestingly, no comparable enrichment of α/β-hydrolase genes was observed in Arm024. This result may partially explain the weaker transcriptional and phenotypic responses of the non-symbiotic strain to GR24. However, it should be emphasized that the absence of enrichment does not necessarily indicate the absence of SL perception mechanisms. Alternative signaling pathways, including G-protein-mediated signaling cascades, may also participate in fungal responses to SLs. Therefore, functional validation through gene knockout, heterologous expression, ligand-binding assays, and biochemical characterization will be necessary to determine the precise mechanisms of SL perception in *Armillaria*.

Taken together, our results suggest that the ability to perceive and respond to strigolactones may represent an important adaptive trait associated with symbiosis between *Armillaria* and *Gastrodia elata*. Enhanced responsiveness to host-derived SL signals could facilitate fungal recruitment, rhizomorph proliferation, and symbiotic establishment. Nevertheless, the downstream signaling network linking SL perception to fungal development remains largely unknown and warrants further investigation.

## Conclusion

This study systematically investigated the effects of the synthetic strigolactone analog GR24 on two *Armillaria* species with contrasting symbiotic abilities. GR24 significantly promoted rhizomorph branching and biomass accumulation in the Gastrodia-symbiotic strain *A. gallica* Arm016, whereas only limited effects were observed in the non-symbiotic strain *A. ostoyae* Arm024. Transcriptome analysis revealed extensive transcriptional reprogramming in Arm016, involving genes associated with growth, metabolism, transport, and filamentous development, indicating that strigolactone signaling contributes to fungal growth regulation.

Furthermore, three candidate *α*/*β*-hydrolase proteins were identified in Arm016, and structural analyses suggested that one candidate protein (i2) shares conserved domain architecture and three-dimensional folding characteristics with the canonical plant strigolactone receptor D14. These findings provide preliminary evidence for the existence of D14/KAI2-like strigolactone perception mechanisms in *Armillaria*.

Overall, this study demonstrates that responsiveness to strigolactones differs substantially between symbiotic and non-symbiotic *Armillaria* species and highlights a potential role for SL signaling in regulating fungal development and symbiotic adaptation. Future studies combining genetic, biochemical, and functional approaches will be required to verify the receptor function of candidate proteins and to elucidate the molecular signaling network underlying strigolactone-mediated communication in the *Gastrodia–Armillaria* symbiosis.

## Data Availability

Raw data have been uploaded to the National Center for Biotechnology Information (NCBI) database under SRA project accession number PRJNA759758.

## Footnotes

1
http://www.novogene.com

2
http://wego.genomics.org.cn/

3
https://www.rcsb.org/
